# Efficacy and safety evaluation of acupuncture in the treatment of impaired glucose regulation: A protocol for systematic review and meta-analysis

**DOI:** 10.1097/MD.0000000000027934

**Published:** 2021-12-17

**Authors:** Jiabao Sun, Gaofeng Wang, Xiaoyu Zhi, Xuewei Zhao, Weichen Sun, Yunjie Chu, Xingquan Wu

**Affiliations:** a Department of Acupuncture and Tuina, Changchun University of Chinese Medicine, Changchun, China; b The Affiliated Hospital of Changchun University of Chinese Medicine, Changchun, China; c Beihua University, Jilin, China.

**Keywords:** acupuncture, impaired of glucose regulation, protocol, systematic review

## Abstract

**Background:**

Impaired of glucose regulation belongs to the stage of prediabetes, which is a state of glucose metabolism between diabetes and normal blood glucose. The prevalence of prediabetes in people over 20 years old in China is significantly higher than that in diabetic patients. If no measures are taken to prevent the transition from prediabetes to diabetes, the number of diabetic patients in China will further increase. This study conducted a meta-analysis of the effectiveness of acupuncture in the treatment of impaired glucose regulation by collecting relevant literatures.

**Methods:**

Nine electronic databases: PubMed, EMBASE, Cochrane library, Web of Science, Google Scholar, China National Knowledge Infrastructure, Chinese Biomedical Literature Database, Chinese Scientific and Journal Database, Wan Fang database, and 2 clinical trials register platforms: Chinese Clinical Trial Registry, ClinicalTrials.gov (www.ClinicalTrials.gov/) will be searched for randomized clinical trails of acupuncture for impaired glucose regulation. The screening process will be developed by 2 independent reviewers, and meta-analysis will be performed with RevMan (V5.3.5) software.

**Results:**

This meta-analysis further confirmed the benefits of acupuncture in the treatment of impaired of glucose regulation.

**Conclusion:**

This study will provide a high-quality evidence of the efficacy and safety of acupuncture on patients with impaired glucose regulation.

**PROSPERO registration number:**

INPLASY202170058.

**Ethics and dissemination:**

This systematics review will evaluate the efficacy and safety of acupuncture in the treatment of impaired of glucose regulation. Since all the data included were published, the systematic review did not require ethical approval.

## 1 Introduction

As a chronic disease highly related to heredity, environment, living habits, and other factors, diabetes poses a serious threat to human health.^[1–4]^ Impairment of glucose regulation belongs to the stage of prediabetes, which is a state of glucose metabolism between diabetes and normal blood glucose, including 3 abnormal states: impaired glucose tolerance alone, impaired fasting glucose alone, and impaired fasting glucose combined with impaired glucose tolerance.^[5]^ The Diabetes Prevention and Treatment Guidelines issued by the Diabetes Society of the Chinese Medical Association in 2020 indicated that the prevalence of prediabetes (15.5%) was significantly higher than that of diabetes (9.7%) in people over 20 years old in China.^[6–8]^ The prevalence of prediabetes in people over 20 years old in China is significantly higher than that in diabetic patients. If no measures are taken to prevent the transition from prediabetes to diabetes, the number of diabetic patients in China will further increase.^[9–11]^

At present, the treatment of patients with prediabetes in China mainly focuses on diet control and exercise to reduce the risk of diabetes.^[12–15]^ Acupuncture, as a traditional external treatment method, is widely used as adjuvant therapy. Therefore, this study conducted a meta-analysis of the effectiveness of acupuncture in the treatment of impaired glucose regulation by collecting relevant literatures.

## 2 Method and design

We will conduct a system review according to the preferred report items declared by the system review and meta analysis program in 2015.^[16]^

### 2.1 Study inclusion criteria

We will include randomized controlled trials related to acupuncture for impaired glucose regulation. There are no restrictions on the language or status of publication. Patients of all ages, genders and ethnicities with impaired glucose regulation will be included.

#### 2.1.1 Participants

Patients diagnosed with impaired glucose regulation included impaired fasting glucose, impaired glucose tolerance, and impaired fasting glucose combined with impaired glucose tolerance. Impair of fasting blood glucose: fasting venous plasma glucose ≥5.6 mmol L^–1^ (100 mg DL^–1^) and <7.0 mmol L^–1^ (126 mg DL^–1^); Intravenous plasma glucose level was less than 7.8 mmol L^–1^ (140 mg DL^–1^) 2 hour after loading. Impaired glucose tolerance: Intravenous plasma glucose ≥7.8 mmol L^–1^ (140 mg DL^–1^) and <11.1 mmol L^–1^ (200 mg DL^–1^) at 2 hour after load, and fasting venous plasma glucose <7.0 mmol L^–1^ (126 mg DL^–1^).^[8]^

#### 2.1.2 Interventions

The treatment plan of the experimental group was limited to acupuncture treatment, and there was no restriction on the type or method of acupuncture.

#### 2.1.3 Comparisons

The control group did not receive any acupuncture treatment (western medicine, Chinese medicine, placebo or conventional treatment).

#### 2.1.4 Outcomes

Results include effectiveness and safety. Validity indicators include primary outcome indicators and secondary outcome indicators. The primary outcome measures were diagnostic criteria, including: fasting venous plasma glucose level and 2 hour post-load venous plasma glucose level; Clinical total effective rate = (cured number + effective number)/total number × 100%; Secondary outcome measure: TCM syndrome scale score. Safety refers to the incidence of adverse events (bleeding, pain, hematoma, syncope, etc).

### 2.2 Search methods

We will search 9 electronic databases of PubMed, EMBASE, Cochrane Library, Web of Science, China National Knowledge Infrastructure, Chinese Biomedical Literature Database, Chinese Scientific and Journal Database and Wan Fang database to identify literature of randomized clinical trails of acupuncture for impaired sugar regulation. Besides, we will also search Chinese Clinical Trial Registry and ClinicalTrials.gov (www.ClinicalTrials.gov/) for in-progress trials with unpublished data. Table 1 shows PubMed search strategy.

**Table 1:** Search strategy for PubMed.

No	Search terms
#1	Acupuncture. ti,mesh.
#2	Acupuncture therapy. ti,ab.
#3	Acupoint. ti,ab.
#4	Or #1-#3
#5	Impaired glucose regulation. ti,ab.
#6	IGR. ti,ab
#7	Or #4-#5
#8	Randomized controlled trial. pt.
#9	Controlled clinical trial. pt.
#10	Randomized. ab.
#11	Randomly. ab.
#12	Trial. ab.
#13	Or #8-#12
#14	Exp animals/not humans. sh.
#15	#13 not #14
#16	#4 and #7 and #15

IGR = impaired of glucose regulation.

### 2.3 Data collection and management

#### 2.3.1 Selections of studies

**Figure 1:**
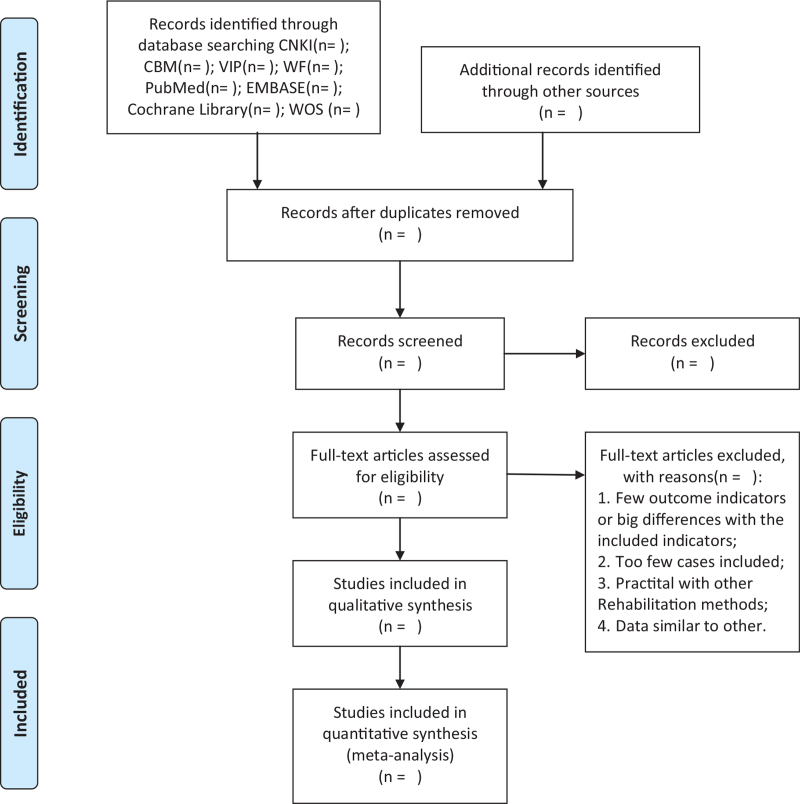
PRISMA flow diagram of study and exclusion. CBM = Chinese Biomedical literature Database, CNKI = China National Knowledge Infrastructure, development and evaluation reliability study, EMBASE = Embase, PRISMA = preferred reporting items for systematic reviews and meta analyses, VIP = Chinese Scientific and Journal Database, WF = Wan Fang, WOS = web of science.

Two reviewers (XYZ and XWZ) will conduct studies selecting. First, they will eliminate duplicate articles with EndNote software (V. X9.0, Thomson ResearchSoft, Stanford, America), they will screen literature with inclusion and exclusion criteria independently. Afterward, through reading titles and abstracts, literature that is obviously not applicable will be deleted. Finally, included articles will be chosen by screening the full articles. (Fig. 1 shows the screening process). All screening procedures will be undertaken by 2 researchers independently. Disagreements of decision making will be solved by referring to the third reviewer (XQW).

#### 2.3.2 Data extraction

Two reviewers (XYZ and XWZ) will select literature and extract data in accordance with the retrieval strategy. Title of the study, first author's name, publication year, journal; information of participants: gender, age, study design, sample size, diagnosis standard, intervention, and outcome indicators will be extracted from the included studies with a standardized form for extracting data by the 2 reviewers independently from the included studies, they will also crosscheck the results, disagreements will be solved by referring to a third reviewer (YJC).

### 2.4 Risk of bias assessment

Two independent reviewers (GFW and JBS) will assess the risk of bias with Cochrane Risk of Bias Tool according to the Cochrane Handbook 5.1.0 for Systematic Reviews of Interventions. The 2 reviewers will assess 7 items, which consist of the risk of bias of sequence generation, allocation concealment, blinding of participants personnel and outcome assessment, incomplete outcome data, selective outcome reporting, and other bias. If there is disagreement during the assessing process, 2 reviewers will discuss or consult the third reviewer (WCS) for a decision. Three evaluation grades are low, unclear, and high risk of bias.

### 2.5 Measures of treatment effect

To assess acupuncture in the treatment of impaired sugar regulation. Pooled risk ratio with 95% credible intervals will be used for investigating dichotomous variables. Standard mean differences with 95% credible intervals or weighted mean differences will be chosen for analyzing continuous variables.

### 2.6 Dealing with missing data

We will e-mail the corresponding author to obtain the necessary information, which is missing or insufficient. If failed, the analysis will be conducted based on the available studies, and we will review the potential impact of missing information.

### 2.7 Assessment of heterogeneity

*I*^2^ will be used for assessing statistical heterogeneity. It is acknowledged that *I*^2^ < 25% indicates negligible heterogeneity, 25% < *I*^2^ < 50% indicates mild heterogeneity, 50% < *I*^2^ < 75% moderate heterogeneity, and *I*^2^ ≥ 75% high heterogeneity.

### 2.8 Assessment of reporting bias

Over 10 studies included,^[17]^ we will take advantage of funnel plot to assess the reporting bias. Symmetrical funnel indicates no publishing bias, but if the funnel is not symmetrical, which indicates publishing bias exists. *P* value will be utilized, while less than 10 studies included.

### 2.9 Data syntheses

We will take advantage of RevMan software (version 5.3.5, The Cochrane Collaboration, Oxford, England) for Statistical analyses performing. Only if there is no or mild significant heterogeneity (*I*^2^ < 50%; *P* > .1), we will apply the fixed-effect model, or the random-effects model will be selected.

### 2.10 Analysis of subgroups or subsets

If there exists potential heterogeneity, we will perform subgroup analysis based on methods of treatment, gender, age, or other items.

### 2.11 Sensitivity analysis

Robustness of the results will be assessed by sensitivity analysis performance which will focus on the processing method of missing data.

### 2.12 Grading the quality of evidence

Grading of recommendations assessment, development, and evaluation reliability study will be implemented to assess the quality of evidence. There are 4 levels of results: very low, low, moderate, and high.

### 2.13 Ethics and dissemination

Due to nothing of the information will be obtained from an individual participant, the systematic review does not need ethical approval.

## 3 Discussion

To our knowledge, there has not been a systematic review nor meta-analysis about acupuncture for Impaired sugar regulation. Acupuncture are traditional Chinese external medical treatments, which have been used in China for thousands of years. A meta-analysis of 1943 patients with T2DM showed that acupuncture can reduce blood glucose, improve body weight, and improve insulin sensitivity.^[18]^ With impaired glucose regulation as a prediabetic, acupuncture should be more effective.

Therefore, we will systematically review the efficacy of acupuncture in adjuvant therapy of impaired glucose regulation, and provide new strategies for clinical application.

## Author contributions

Jiabao Sun and Xingquan Wu had the original idea of this work and drafted the protocol. The search strategy was developed by all the authors and will be performed by Jiabao Sun, Gaofeng Wang, Xiaoyu Zhi, Xuewei Zhao, Weichen Sun et al. Xingquan Wu proposed some advice for design and revision. Xiaoyu Zhi, Xuewei Zhao, and Yunjie Chu independently collected and extracted the eligible studies. Jiabao Sun, Gaofeng Wang, and Weichen Sun assessed the bias risk and dealt with missing data. All the authors participated in this study critically revised the final version of the manuscript and confirmed the publication of this protocol.

**Conceptualization:** Jiabao Sun, Xingquan Wu.

**Data curation:** Xiaoyu Zhi, Xuewei Zhao, Yunjie Chu.

**Formal analysis:** Jiabao Sun, Gaofeng Wang, Weichen Sun.

**Funding acquisition:** Yunjie Chu.

**Investigation:** Xiaoyu Zhi, Xuewei Zhao.

**Methodology:** Jiabao Sun, Gaofeng Wang.

**Supervision:** Xingquan Wu.

**Validation:** Jiabao Sun, Gaofeng Wang, Xingquan Wu.

**Writing – original draft:** Jiabao Sun

**Writing – review & editing:** Jiabao Sun, Yunjie Chu, Xingquan Wu.
